# Pilot investigation on the dose-dependent impact of irradiation on primary human alveolar osteoblasts in vitro

**DOI:** 10.1038/s41598-021-99323-8

**Published:** 2021-10-06

**Authors:** Anna-Klara Amler, Domenic Schlauch, Selin Tüzüner, Alexander Thomas, Norbert Neckel, Ingeborg Tinhofer, Max Heiland, Roland Lauster, Lutz Kloke, Carmen Stromberger, Susanne Nahles

**Affiliations:** 1 Cellbricks GmbH, Berlin, Germany; 2 Department of Medical Biotechnology, Technische Universität Berlin, Berlin, Germany; 3 Department of Oral and Maxillofacial Surgery, Charité-Universitätsmedizin Berlin, Corporate Member of Freie Universität Berlin, Humboldt Universität zu Berlin, Berlin, Germany; 4 Department of Radiation Oncology, Charité-Universitätsmedizin Berlin, Corporate Member of Freie Universität Berlin, Humboldt-Universität zu Berlin, Berlin, Germany; 5 German Cancer Consortium (DKTK) Partner Site Berlin, Berlin, Germany

**Keywords:** Head and neck cancer, Preclinical research

## Abstract

Radiotherapy of head and neck squamous cell carcinoma can lead to long-term complications like osteoradionecrosis, resulting in severe impairment of the jawbone. Current standard procedures require a 6-month wait after irradiation before dental reconstruction can begin. A comprehensive characterization of the irradiation-induced molecular and functional changes in bone cells could allow the development of novel strategies for an earlier successful dental reconstruction in patients treated by radiotherapy. The impact of ionizing radiation on the bone-forming alveolar osteoblasts remains however elusive, as previous studies have relied on animal-based models and fetal or animal-derived cell lines. This study presents the first in vitro data obtained from primary human alveolar osteoblasts. Primary human alveolar osteoblasts were isolated from healthy donors and expanded. After X-ray irradiation with 2, 6 and 10 Gy, cells were cultivated under osteogenic conditions and analyzed regarding their proliferation, mineralization, and expression of marker genes and proteins. Proliferation of osteoblasts decreased in a dose-dependent manner. While cells recovered from irradiation with 2 Gy, application of 6 and 10 Gy doses not only led to a permanent impairment of proliferation, but also resulted in altered cell morphology and a disturbed structure of the extracellular matrix as demonstrated by immunostaining of collagen I and fibronectin. Following irradiation with any of the examined doses, a decrease of marker gene expression levels was observed for most of the investigated genes, revealing interindividual differences. Primary human alveolar osteoblasts presented a considerably changed phenotype after irradiation, depending on the dose administered. Mechanisms for these findings need to be further investigated. This could facilitate improved patient care by re-evaluating current standard procedures and investigating faster and safer reconstruction concepts, thus improving quality of life and social integrity.

## Introduction

Multi-disciplinary treatment with curative (chemo)radiotherapy (RT) is recommended in patients with locally advanced head and neck squamous cell carcinoma (HNSCC)^1^. Dental assessment and stabilizing oral health before RT are crucial as dental extraction or invasive interventions following RT might be risk factors for osteoradionecrosis of the jaw, a serious late side effect after RT treatment^2–4^. The conservative course of action for dental rehabilitation is to place enosseal implants 6 months after chemotherapy and radiation treatment at the earliest. This prolongs deficits in oral health and social integrity, which play an important role in the quality of life of patients with HNSCC^5,6^. Profound knowledge of the effects of radiation on human alveolar bone cells is still lacking, though it is decisive to plan dental implant concepts after the therapy. Neckel et al.^7^ showed that the radiation dose to the specific area of the implants can be seen as a relevant risk factor for peri-implant tissue health and therefore long-term survival.

The in vivo situation in the irradiated jaw is highly complex due to the interplay of the various cell types present in the alveolar bone, the surrounding soft tissue and the microbiome. Possible mechanisms of radiation-induced late damages on mandibular bone are hypovascularization, and a reduction of osteoblast numbers, osteoblastic activity and bone regeneration^8–11^. Osteoblasts have been repeatedly reported to be a major contributor to the decrease in bone density after irradiation^12–15^. Previous in vitro studies have relied on osteoblastic murine cell lines like MC3T3-E1 or human fetal cell lines from skeletal bones^16–21^. These models can only partially mimic the in vivo situation due to interspecies differences and the fact that alveolar osteoblasts behave differently than those derived from long bones, for example regarding their proliferation, mineralization and angiogenic properties^22,23^.

This is the first study employing primary human alveolar osteoblasts to investigate the impact of irradiation on this cell type, which is essential for the regeneration of bone. Human alveolar osteoblasts were irradiated with 2, 6, and 10 Gy and compared to non-irradiated controls regarding their proliferation, morphology, mineralization, and expression of marker genes and proteins.

## Results

### Impact of irradiation on the proliferation

**Figure 1:**
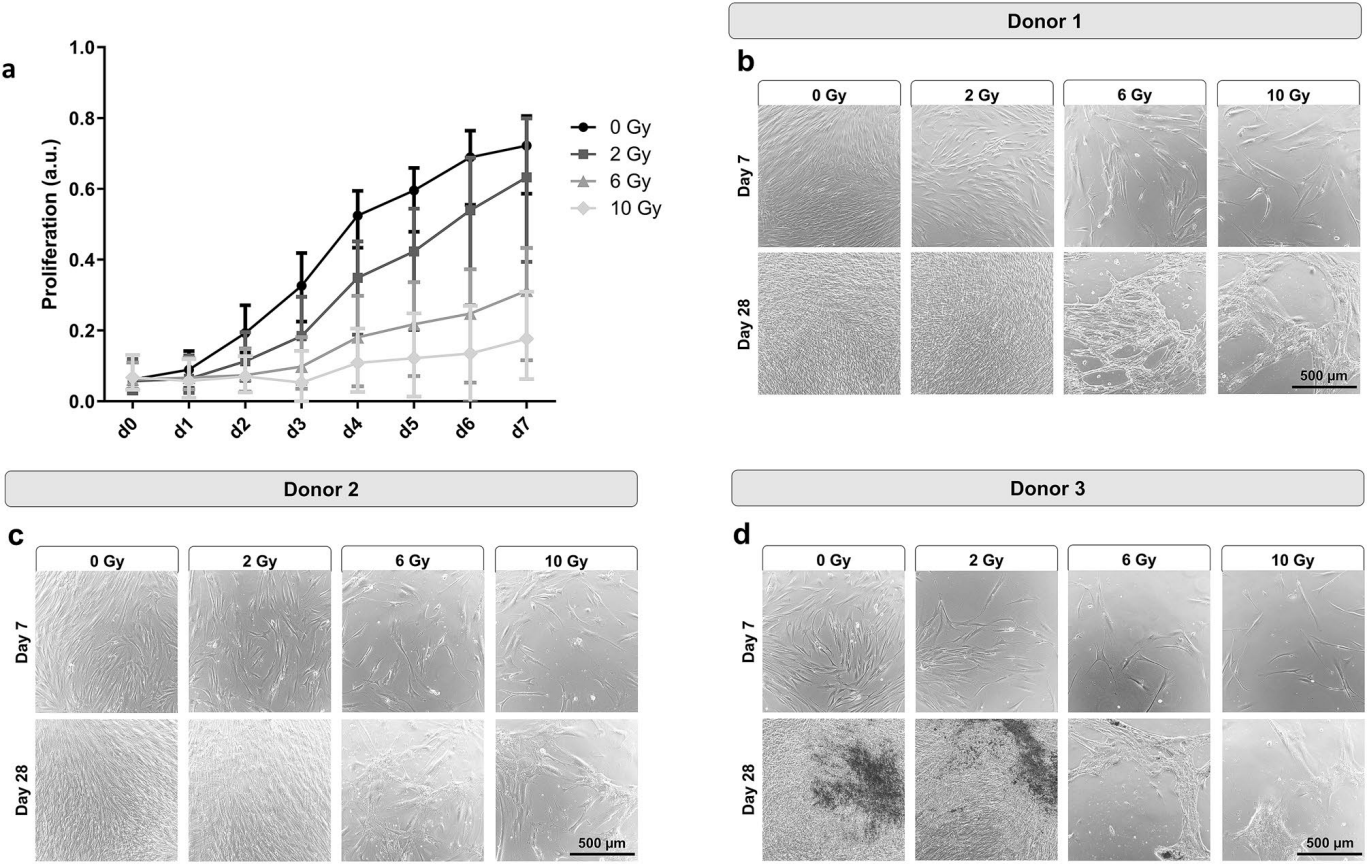
Impact of irradiation on the proliferation of JHOBs. (**a**) Proliferation is inversely proportional to the MFI of the CellTrace Violet dye. JHOBs were stained with CellTrace Violet, seeded, and irradiated the next day. Sampling was performed every day and cells were analyzed using flow cytometry. Proliferation index is calculated as the inverse normalized MFI of CellTrace. Data are presented as mean ± s.d. n = 3 donors, where three biological replicates were analyzed for each donor. (**b**–**d**) Microscopic images of JHOBs on day 7 and 28 after irradiation.

Irradiation of primary jawbone-derived human osteoblasts (JHOBs) decreased but did not completely abolish the proliferation over the course of the experiment, as indicated by the proliferation index (PI) (Fig. 1a). In general, a higher radiation dose resulted in a flatter slope of the curve (P_0 Gy vs. 2/6/10 Gy_ < 0.0001, P_2 Gy vs. 6/10 Gy_ < 0.0001, P_6 Gy vs. 10 Gy_ = 0.0011, Supplementary Table S1). Looking at individual donors, all three JHOB cultures showed a dose-dependent decrease in proliferation after irradiation, with the cell culture from donor 3 presenting overall a significantly lower PI (Supplementary Fig. S1a–c; P_donor 1 vs. 3_ = 0.0011, P_donor 2 vs. 3_ < 0.0001, Supplementary Table S2). Proliferation of cells irradiated with 2 Gy was lower compared to untreated cells, although differences were small and only significant for donor 3, but not donor 1 and 2 (P_donor 1_ = 0.9519, P_donor 2_ = 0.7793, P_donor 3_ = 0.0219). In contrast, treatment with 6 and 10 Gy significantly inhibited the proliferative activity compared to the non-irradiated controls for all donors (Supplementary Table S3). A significant difference of the effect of irradiation on the JHOBs was determined between the irradiation conditions for all combinations, except for donor 1, 2 and 6 Gy (P = 0.077), and donor 2, 6 and 10 Gy (P = 0.2589).

Monolayer cultures of JHOBs from donor 3 were less confluent after 7 days of cultivation compared to the other donors (Fig. 1b–d, top lanes). JHOBs irradiated with 6 or 10 Gy were not able to form a confluent monolayer after 28 days of cultivation, while 2 Gy conditions were qualitatively not discernible to 0 Gy on day 28 (Fig. 1b–d, bottom lanes).

### Impact of irradiation on the mineralization

**Figure 2:**
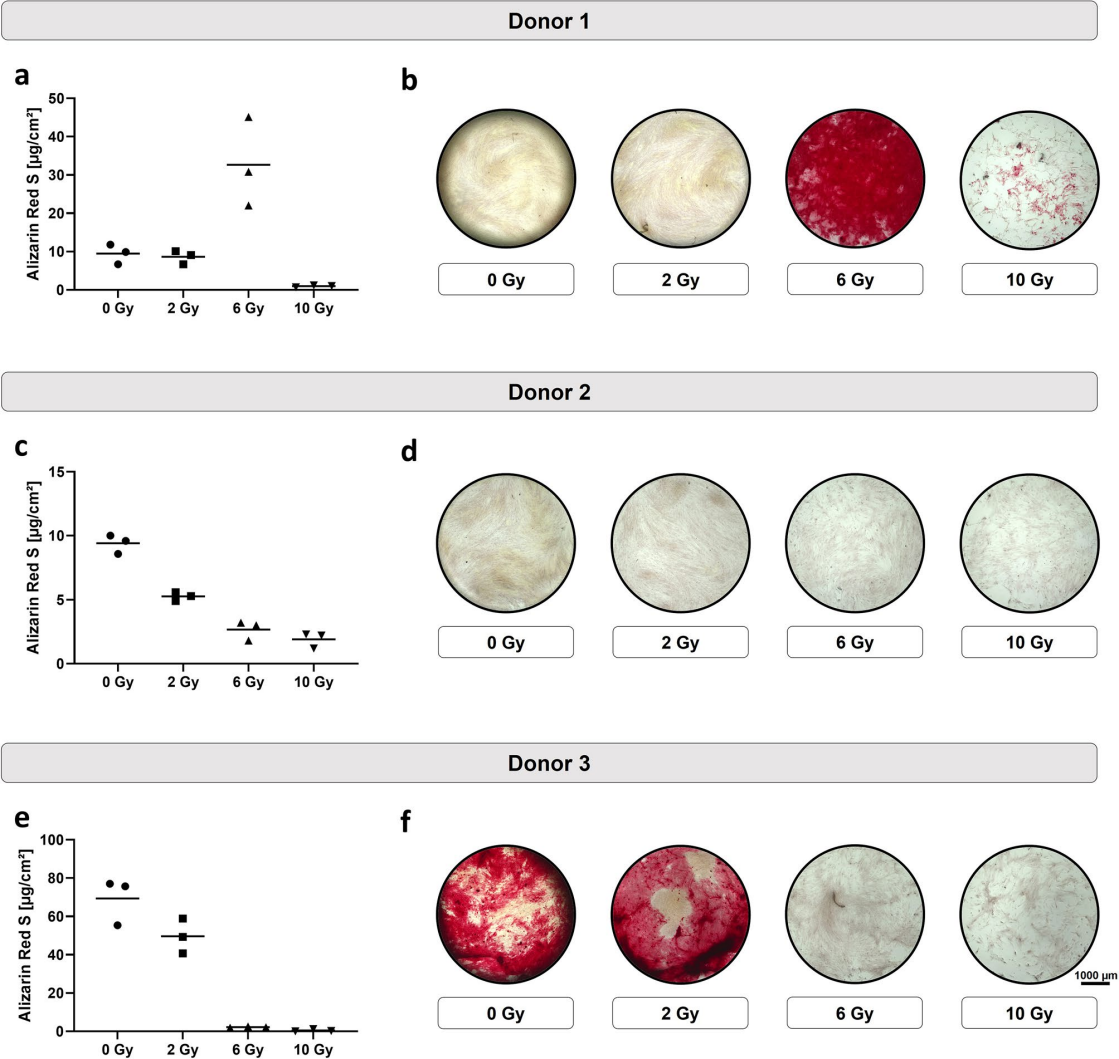
Mineralization of irradiated JHOBs. JHOBs were treated with different radiation doses and the level of mineralization was quantitatively (**a**, **c**, **e**) and qualitatively (**b**, **d**, **f**) assessed after 28 days of cultivation by Alizarin Red S staining. n = 3 biological replicates for each donor.

The impact of different irradiation doses on the mineralization varied between the donors. For donor 1, strong mineralization was only observed for cells treated with 6 Gy (Fig. 2a,b), whereas low mineralization was observed for the other conditions. Donors 2 and 3 displayed similar patterns, where lower mineralization correlated with higher radiation doses (Fig. 2c,e). However, mineralization levels were overall very low for donor 2 as clearly recognizable in the staining images (Fig. 2d,f).

### Impact of irradiation on the protein expression

**Figure 3:**
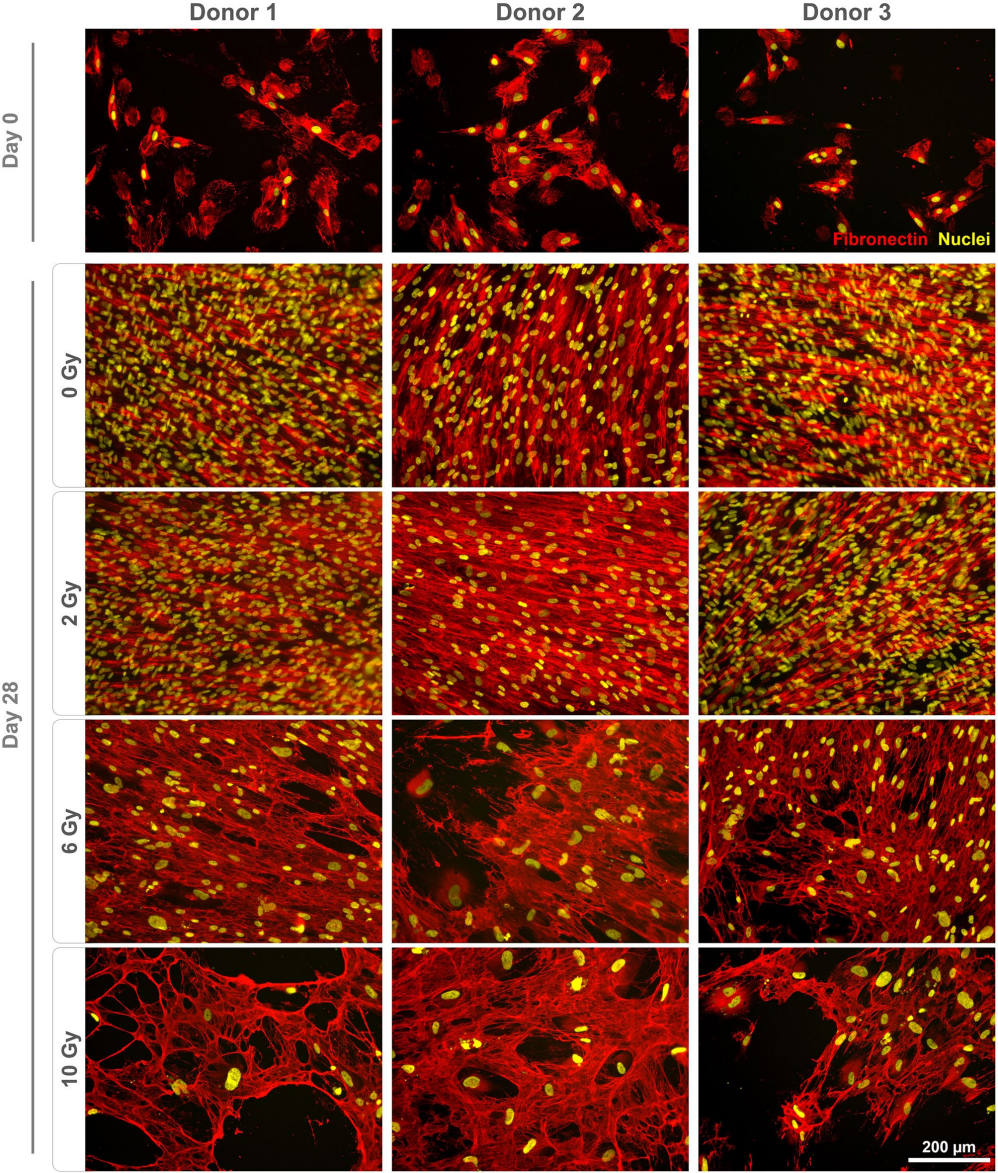
Immunostaining of irradiated JHOBs for fibronectin. Cells were irradiated with 2, 6 and 10 Gy or sham, and stained for expression of fibronectin (red) after 28 days of cultivation in osteogenic medium. Non-treated cells on day 0 were used as a control. Nuclei were counterstained with DAPI (yellow).

**Figure 4:**
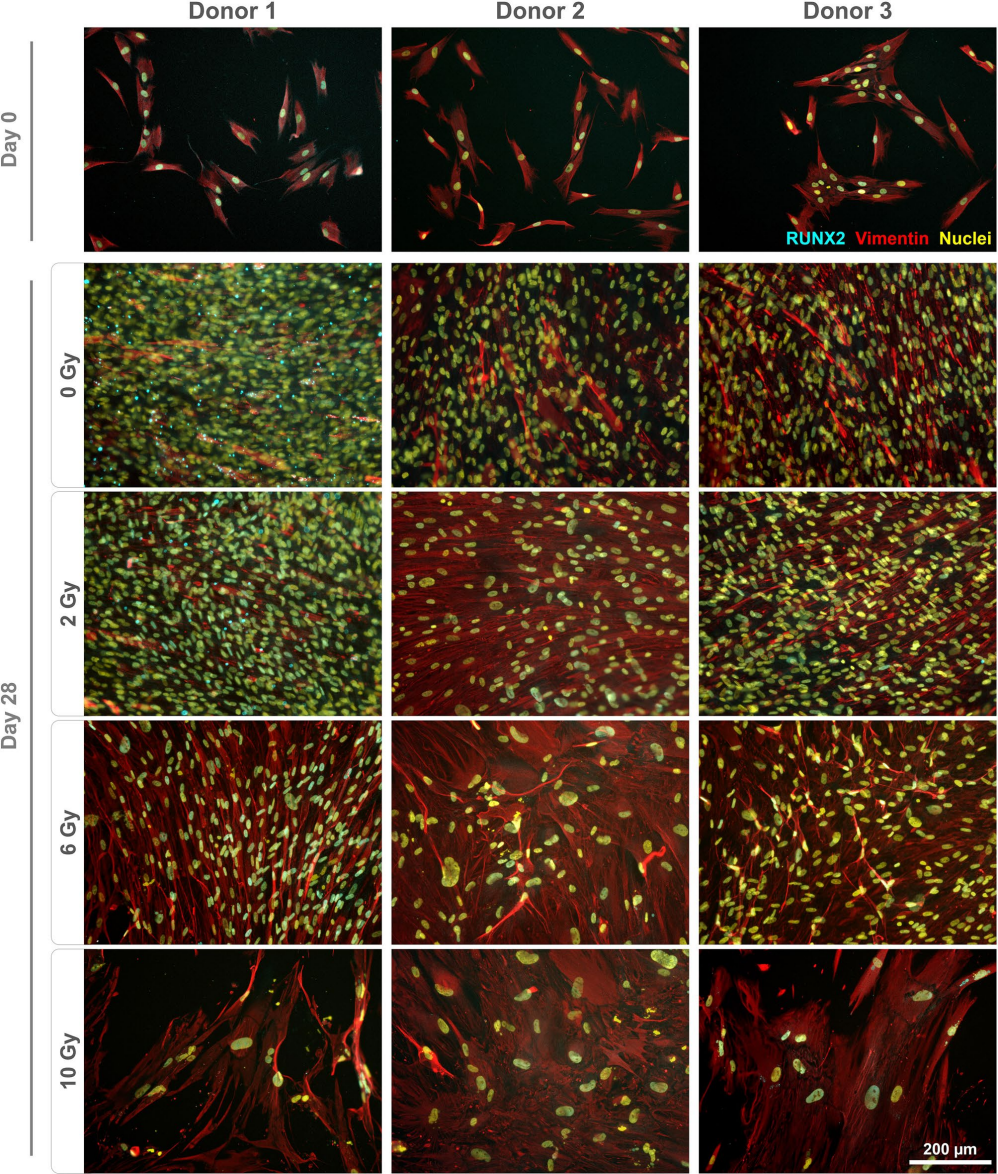
Immunostaining of irradiated JHOBs for RUNX2 and vimentin. Cells were irradiated with 2, 6 and 10 Gy or sham, and stained for expression of RUNX2 (cyan) and vimentin (red) after 28 days of cultivation in osteogenic medium. Non-treated cells on day 0 were used as a control. Nuclei were counterstained with DAPI (yellow).

**Figure 5:**
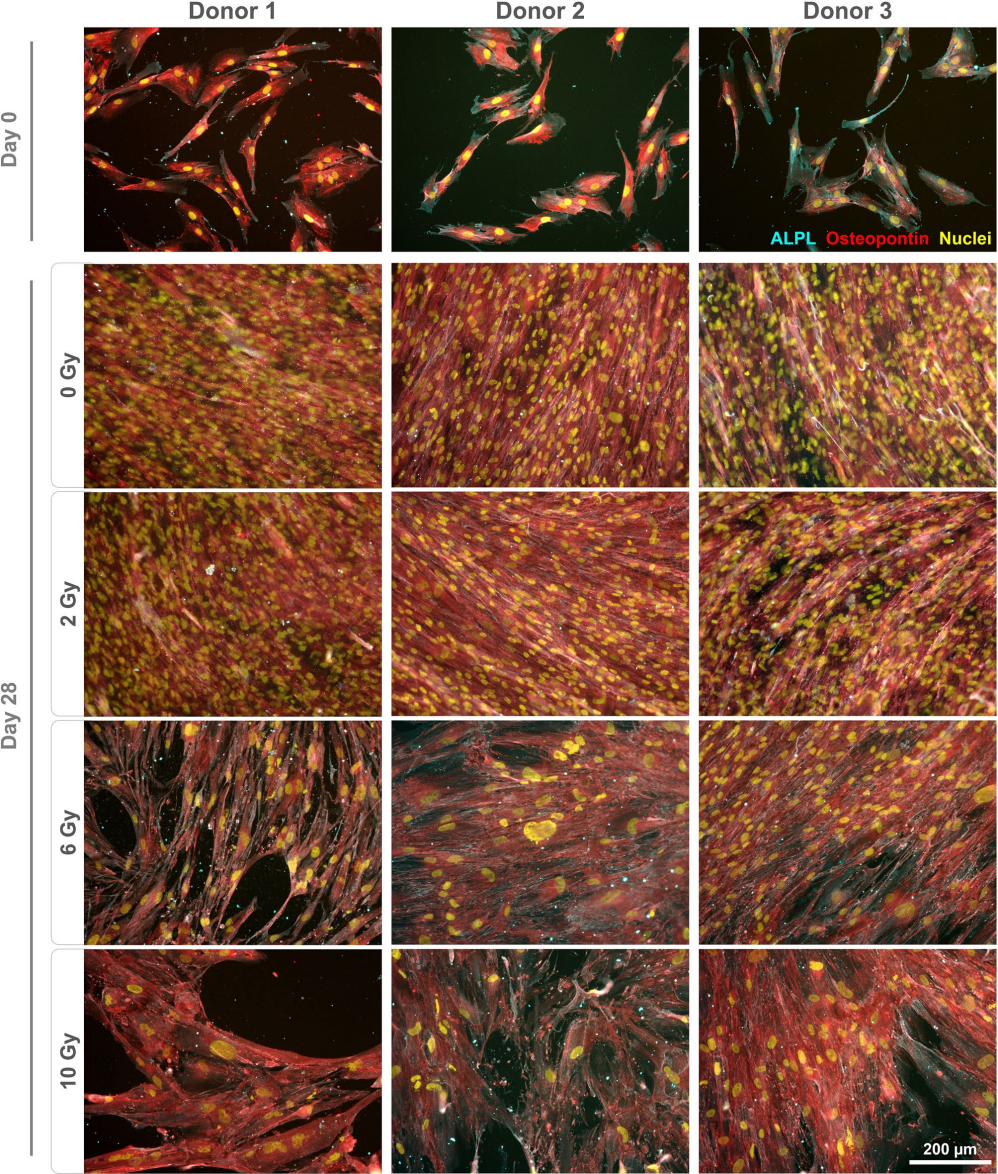
Immunostaining of irradiated JHOBs for ALPL and osteopontin. Cells were irradiated with 2, 6 and 10 Gy or sham, and stained for expression of ALPL (cyan) and osteopontin (red) after 28 days of cultivation in osteogenic medium. Non-treated cells on day 0 were used as a control. Nuclei were counterstained with DAPI (yellow).

**Figure 6:**
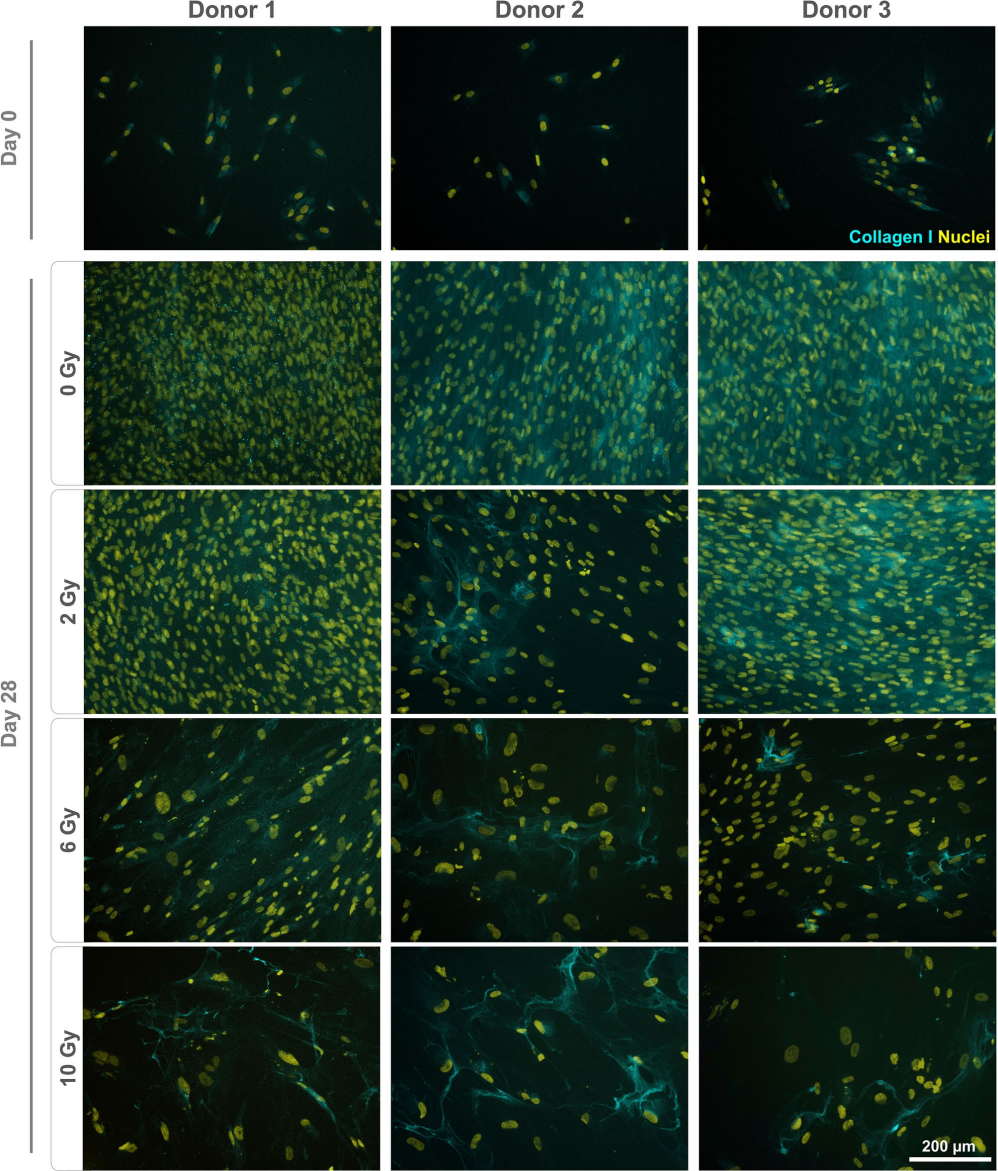
Immunostaining of irradiated JHOBs for collagen I. Cells were irradiated with 2, 6 and 10 Gy or sham, and stained for expression of collagen I (cyan) after 28 days of cultivation in osteogenic medium. Non-treated cells on day 0 were used as a control. Nuclei were counterstained with DAPI (yellow).

Monolayers were stained to qualitatively assess marker gene expression on the protein level. Fibronectin expression was found to be altered following irradiation (Fig. 3). For all donors, non-irradiated and cells irradiated with 2 Gy displayed a dense, well-organized network. In contrast, the staining pattern for fibronectin appeared different in cultures treated with 6 and 10 Gy, coinciding with the presence of enlarged nuclei. Staining for vimentin revealed an altered morphology with irregular shapes and expanded cell bodies for cells treated with 6 and 10 Gy (Fig. 4). Expression of RUNX2 (Fig. 4), ALPL, osteopontin (Fig. 5) and osteonectin (Supplementary Fig. S2) was demonstrated for all conditions, with no discernible changes of the staining patterns besides the enlargement of the cells. Collagen I was found to be expressed in the cytoplasm on day 0, while extensive staining of the well was found for 0 and 2 Gy conditions (except for donor 2). A less homogenous deposition of collagen I was visible in 6 and 10 Gy conditions (all donors), and also in the 2 Gy condition of donor 2 (Fig. 6). No unspecific staining of secondary antibodies was detected (Supplementary Fig. S3).

### Impact of irradiation on the marker gene expression

**Figure 7:**
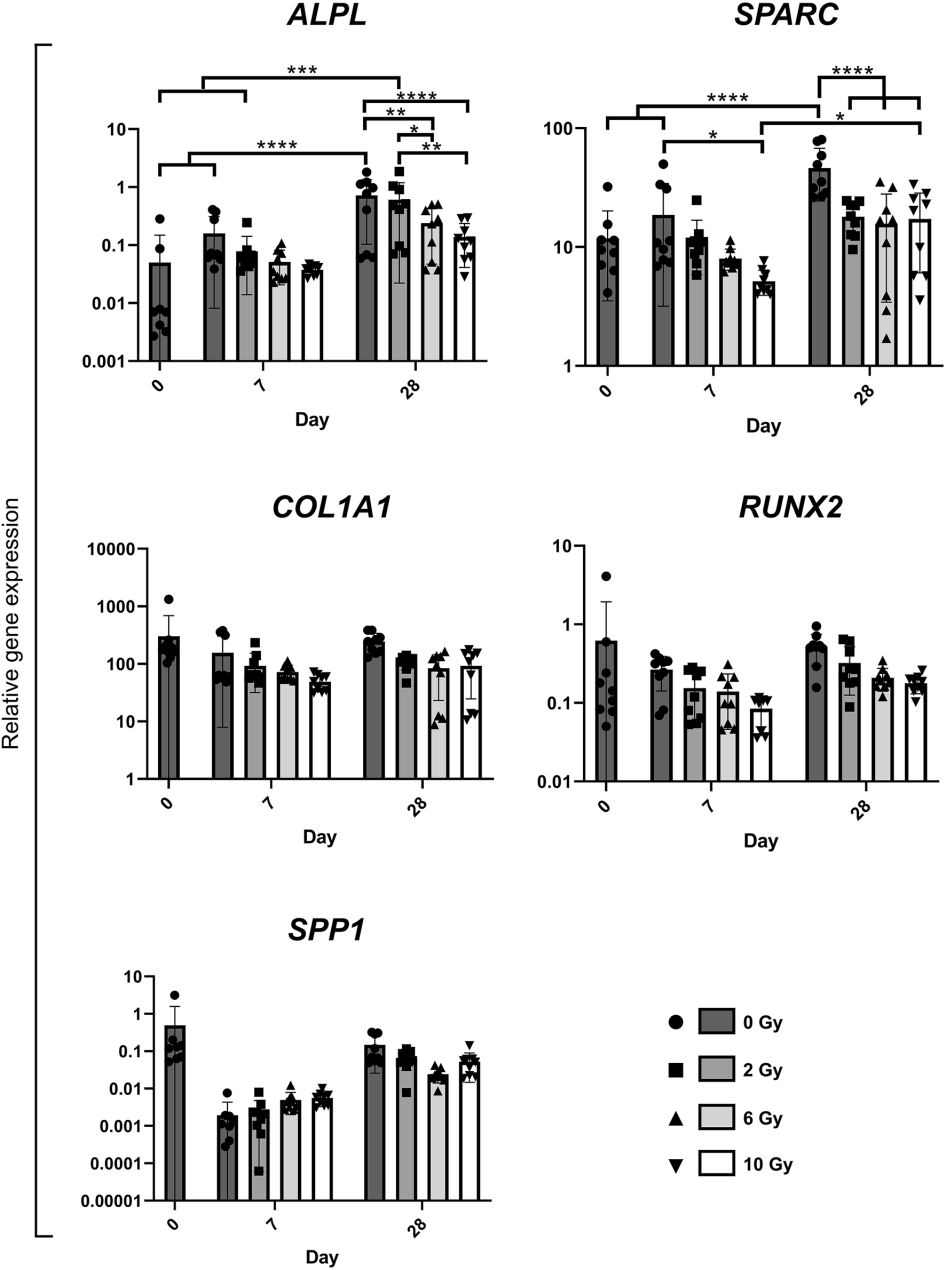
Marker gene expression of irradiated JHOBs. Relative gene expression of the osteoblast differentiation markers ALPL, COL1A1, RUNX2, SPARC, and SPP1 were cultivated in osteogenic medium and analyzed on days 0, 7 and 28. Expression was normalized to UBE2D2 expression. * = P < 0.05. ** = P < 0.01. *** = P < 0.001. **** = P < 0.0001. Data are presented as mean ± s.d. n = 3 donors, where three biological replicates were analyzed for each donor.

Expression of *ALPL* was upregulated for non-irradiated controls (P < 0.0001) and upon irradiation with 2 Gy (P = 0.0002) (Fig. 7). After 28 days of cultivation, a significant downregulation upon irradiation with 6 and 10 Gy was detected compared to a 2 Gy dose and non-irradiated controls (Supplementary Table S4). A significant increase of *SPARC* expression was observed over the course of the experiment in non-irradiated cultures (P < 0.0001). Here, irradiation resulted in a decrease on day 7 which was significant for an irradiation with 10 Gy (P = 0.0375). On day 28, expression levels were significantly lowered for all doses (P < 0.0001).

Expression levels of *COL1A1* and *RUNX2* appeared stable for non-irradiated cells over the course of the experiment. Upon irradiation, a tendency for a dose-dependent reduction of the expression was observable. Expression of *SPP1* in non-irradiated cells decreased from day 0 to day 7, followed by an increase to day 28. Following irradiation, the expression levels showed a tendency to slight elevation on day 7, which was inversed on day 28.

## Discussion

Previous studies on the effects of irradiation on osteoblasts have shown contradicting results^21,24–26^. This can be partly explained by the utilization of multiple cell lines, which resemble varying differentiation states^27^. In contrast, this is the first study investigating primary human osteoblasts obtained from the jawbone. Here, results from previous studies were partially confirmed, as detailed below. In this context, interindividual heterogeneities in the response of JHOBs to the irradiation were observed. This indicates the feasibility to differentiate between radiation-insensitive and radiation-sensitive patients, which could enable personalized treatment concepts for dental reconstruction following radiotherapy.

A dose-dependent decrease of the proliferation capacity was demonstrated (Fig. 1), as has been reported before^17,19,25,28^. One possible explanation is a reversible or permanent shift in the cell cycle distribution^16,19,21,26^. Deeper insights into molecular mechanisms are desirable but were beyond the scope of this study. Additionally, a decrease in proliferation has been associated with the induction of differentiation as well as cell destruction^17^. The present data suggest that differentiation of alveolar osteoblasts was not enhanced by irradiation as shown by mineralization and gene expression (Figs. 2 and 7), contradicting previous studies using murine cell lines^17,21,25^.

Several authors have reported a certain threshold below which a few aspects of cell function were recovered^19,21,26^. This was to some extent confirmed in the present study. For instance, confluent monolayers were observed after 28 days of cultivation for cells treated with 2 Gy and controls, but not for 6 and 10 Gy (Fig. 1b–d). Immunostaining demonstrated analogous results since staining patterns for 2 Gy and controls were not distinguishable for most of the markers (Figs. 3, 4, 5). However, interindividual differences were observed as 2 Gy-irradiated cells of donor 2 displayed an altered staining pattern of collagen I, whereas those of donor 1 and 3 did not (Fig. 6). This again underlines the need for a higher number of donors in follow-up studies to allow for more universal conclusions.

Irradiation with 6 and 10 Gy resulted in an altered cell morphology, independent from the donors (Figs. 4, 5, Supplementary Fig. S2). The number of cells was decreased, while cell bodies and nuclei were enlarged, in accordance with observations made by Huang et al.^18^. Immunostaining revealed that the organization of fibronectin, one of the most abundant extracellular matrix (ECM) proteins secreted by the cells^29^, was disrupted, leading to impairment of the ECM (Fig. 3). This can also impact differentiation of osteoblasts, as this process requires interaction with fibronectin via integrin receptors^30^, as well as formation of new bone^29^. Analogously, irradiation with 6 and 10 Gy affected the overall expression of collagen I (Fig. 6), which is the main organic component of the bone matrix^31^, changing from a diffused to a more condensed staining pattern. High irradiation doses might therefore impair the formation of the ECM and alter the bone structure, as has been reported for ORN^32^.

As this is the first pilot study investigating irradiation effects on primary human alveolar osteoblasts, only three donors were examined. As expected based on the observation of a donor-specific behavior of alveolar osteoblasts in previous in vitro and in vivo studies^22,33–35^, differences between donors were observed regarding the impact of irradiation, particularly concerning the mineralization capacity and gene expression. To compensate for interindividual differences and therefore enable more universal conclusions, the number of donors should be increased. Another highly interesting possibility is the investigation of gender-specific differences, as previous studies have reported an impact of the gender on clinical aspects and cell physiology^7,23^.

The existence of heterogenous populations in the ex vivo cultures is another potential explanation for varying results. In general, highly proliferative cells are being more affected by irradiation. For donor 1, administration of 6 and 10 Gy could have led to survival of less proliferative and therefore more differentiated osteogenic cells, resulting in a distinct red staining of the matrix (Fig. 2b), compared to 0 and 2 Gy. In contrast, JHOBs isolated from donor 3 presented an overall more differentiated phenotype, as shown by their lower proliferation (Supplementary Fig. S1c, Supplementary Table S1) and stronger mineralization of non-irradiated cells (Fig. 2e) compared to the other donors. Advanced methods like single cell sequencing and analysis of DNA methylation patterns can help to unravel this.

Differing results between donors can also arise from the origin of the JHOBs, since the bone specimen used for this study were obtained both from mandibular (donor 1) and maxillary (donors 2 and 3) jawbone. Although they share the same developmental origin, their physiologies differ, for example regarding the ratio of cortical and cancellous bone, vascular density, and the size of medullary cavities^36–38^. This could for example explain the dissimilar behavior of cells regarding their mineralization. Here, donor 3 presented a dose-dependent impairment of mineralization, whereas mineralization was strongly increased after irradiation with 6 Gy for donor 1. A separate evaluation of mandible- and maxilla-derived JHOBs in further studies with a higher number of donors per group could elucidate this hypothesis and shed light on whether perceived clinical differences between the upper and lower jaw concerning osteoradionecrosis and implant success really exist.

The transferability of in vitro results to the real multimodal treatment situation for a head and neck cancer patient in vivo is of particular interest. An important factor that needs to be considered is the way of radiotherapy administration. Several authors have pointed out the impact of single dose vs. fractionated radiation, and of the irradiation time point^16,17,24,25^. Furthermore, frequently used photon-based irradiation techniques, proton therapy or a combination of both can be used in head and neck cancer treatment^39^.

In general, the correlation of the in vitro vs. the in vivo dose must be considered^17^. Although this study gives first insights into the radiobiology of human alveolar osteoblasts, the transferability is somehow limited as it is based on a two-dimensional cultivation considering only the investigated cell type. The employment of three-dimensional models like our recently published bioprinted human jawbone model might provide more reliable results than those obtained from conventional 2D culture^40^. Furthermore, the mutual impact of other cell types present in the irradiated bone and soft tissue, including osteoclasts and osteocytes, has to be taken into account to simulate the complex in vivo situation.

Characterising in vitro changes of human alveolar osteoblasts after RT is certainly one step in providing additional information on the mechanism of RT damage and pathogenesis of ORN of the jaw. Furthermore, the different theories by Marx, Delanian and Lefaix propose poor vascularisation as a common feature in the pathogenesis of ORN^41,42^. Vascularity has been studied most frequently in animal models^43–46^, and more recently quantitative research in human mandibular bone found a decrease in the number and density of vessels with higher doses affecting mainly the smaller vessels^47^. Employing co-culture models featuring both endothelial cells and osteoblasts for irradiation studies could to some extent recapitulate this complex interplay and provide further insights into the role of the (micro)vascular system^40,48,49^. Here, JHOBs were investigated detached from other cell types, generating the first data on the impact of irradiation.

## Conclusions

Taken together, this study provides first insights into the dose-dependent effect of irradiation on primary human osteoblasts obtained from the alveolar bone. Further investigations on the underlying molecular mechanisms might reveal potential targets for the prevention, or risk factors for the development of late damages like osteonecrosis. This can eventually contribute to a safer and more predictable patient-specific implant therapy based on the irradiation dose administered for HNSCC therapy, and therefore to an improvement in patient care by refining the timing of implant-support prosthetic rehabilitation.

## Methods

### Cell isolation and culture

Primary jawbone-derived human osteoblasts (JHOBs) were isolated following slightly modified protocols^22,50^. Small bone pieces (approximately 8 mm^3^) of human mandibular (donor 1) or maxillary (donors 2 and 3) jawbone were obtained from healthy donors between 72 and 79 years old during surgery (Informed consent of all participating subjects was obtained, and all methods were performed in accordance with the relevant guidelines and regulations, as approved by Charité’s Ethics Committee, EA4/064/18). Samples were stored at 4 °C in growth medium (high-glucose DMEM supplemented with 10% fetal bovine serum (FBS), 100 IU mL^−1^ penicillin, 100 µg mL^−1^ streptomycin and 2 mM GlutaMAX (Gibco, Thermo Fisher Scientific, Waltham, USA)) until isolation. After removing remaining soft tissue using a scalpel and thoroughly rinsing with phosphate buffered saline (PBS), bone pieces were incubated in Betaisodona iodide solution (Mundipharma, Frankfurt am Main, Germany) for 60 s. Following repeated rinsing with PBS, bone pieces were put in a tissue culture-treated petri dish covered with growth medium. JHOBs were expanded upon confluency around the bone pieces and used at passage 2. All consumables were obtained from Corning Inc. (Corning, USA) unless stated otherwise.

### Irradiation

JHOBs were seeded according to the respective experimental section and allowed to attach to the culture dish overnight. The next day (= day 0), cells were treated with 2, 6, or 10 Gy X-ray radiation dose using the Y.MaxiShot X-ray unit (Yxlon, Hongkong, People's Republic of China; settings: 200 kV, 10 mA, 5.5 FOC). Non-irradiated cells were used as a control (“0 Gy”). Following the radiation procedure, a medium exchange was performed. Live imaging was performed throughout the cultivation period using the BIOREVO BZ-9000 microscope (Keyence, Osaka, Japan).

### Proliferation

To investigate the impact of the radiation dose on the proliferation of JHOBs, cells were stained with CellTrace Violet (Invitrogen, Carlsbad, USA) following the manufacturer’s instructions. Cells were seeded in 12-well plates at 7500 cells cm^−2^ in growth medium with and without the proliferation inhibitor deferoxamine (DFO)^51^. Irradiation of cultured cells was performed the following day (= day 0). Sampling was performed every day until day 7. For this, cells were trypsinized, washed with PBS + 5 mM ethylenediaminetetraacetic acid (EDTA; Corning) + 3% bovine serum albumin (Gibco) and analysed using the MACSQuant Analyzer (Miltenyi, Bergisch Gladbach, Germany) flow cytometer. Data were processed using FlowJo 10 (FlowJo LLC, Ashland, USA). Three wells were analysed per condition and sampling point (n = 3 biological replicates for each donor). Medium was exchanged every other day.

The decrease in fluorescence signal intensity correlates with the proliferative activity of the cells, as the dye is taken up by the cells, gets hydrolyzed and is divided evenly between the daughter cells upon cell division. To compensate for the decrease in fluorescence signal intensity (FI) due to other factors, e.g. photobleaching, cells were cultivated in growth medium supplemented with DFO at 100 µM (Sigma-Aldrich, Saint Louis, USA). Cells were gated for the main population in SSC/FSC and single cell inclusion. Gates for CellTrace Violet-positive cells were set using an unstained control at day − 1. Proliferation index of the cells was calculated as followed: 1 − ((MFI_−DFO, day x_ − MFI_+DFO, day x_)/MFI_day − 1_) with MFI being the median FI of CellTrace-positive cells.

### Mineralization

To analyze the mineralization capacity of JHOBs dependent on the radiation dose, cells were seeded at 10,000 cells cm^−2^ in 12-well or 96-well plates, irradiated as described above and cultivated for 28 days in osteogenic medium (growth medium supplemented with 10 mM β-glycerophosphate (Sigma-Aldrich), 10 nM dexamethasone (AppliChem, Darmstadt, Germany) and 284 µM ascorbic acid phosphate (Sigma-Aldrich)). For every condition and sampling time, three wells were analysed (n = 3 biological replicates for each donor). Cells were fixated using 4% paraformaldehyde (Electron Microscopy Sciences, Hatfield, USA), washed with PBS and distilled water, and stained using 55 mM Alizarin Red S solution (pH 4.1) (C.I. 58005; Carl Roth, Karlsruhe, Germany) for 10 min at room temperature. Stained samples were carefully washed with distilled water, and images were taken using the BIOREVO BZ-9000 microscope (Keyence). Extraction was executed by adding 10% w/v cetylpyridinium chloride (CPC) (Sigma-Aldrich) and incubating for 1 h at room temperature. Absorption was measured at 550 nm and Alizarin Red S was quantified using a standard curve.

### Immunofluorescence

To evaluate expression of marker proteins, an immunofluorescence staining was performed. JHOBs were seeded at 10,000 cells cm^−2^ in 96-well plates and irradiated as described above. Following this, cells were cultivated in osteogenic medium for 28 days. Monolayers were washed with PBS, fixated for 5 min at room temperature using 4% paraformaldehyde and washed with PBS. Following permeabilization with 0.3% Triton X-100 (Alfa Aesar, Ward Hill, USA) at room temperature for 10 min, samples were washed with PBS and blocked with 10% goat serum (Sigma-Aldrich) in PBS for 20 min at room temperature. Primary antibodies, diluted 1:100 in blocking buffer, were incubated at room temperature for 2 h (rabbit anti-RUNX2, mouse anti-alkaline phosphatase, rabbit anti-osteopontin, rabbit anti-fibronectin, rabbit anti-osteonectin (Abcam, Cambridge, United Kingdom); mouse anti-collagen I (Sigma-Aldrich); mouse anti-vimentin (Santa Cruz Biotechnology, Dallas, USA)). For antibody controls, samples were incubated with blocking buffer only. After washing with PBS, samples were incubated with secondary antibodies (goat anti-mouse-CF488 and goat anti-rabbit-CF594 (Biotium, Fremont, USA)), diluted 1:200 in blocking buffer, for 45 min at room temperature, while simultaneously counterstaining with 4′,6-diamidino-2-phenylindole (DAPI; Roche, Basel, Switzerland). After washing three times with PBS, images were taken using the BIOREVO BZ-9000 microscope (Keyence).

### Real-time PCR

To assess the relative expression of bone-specific marker genes on mRNA level, semi-quantitative real-time PCR was performed (see Table 1). For this, JHOBs were seeded at 10,000 cells cm^−2^ in 6-well plates and irradiated the next day as described above (= day 0). Cells were switched to osteogenic medium following the irradiation. Total mRNA of JHOBs was isolated after 0, 7 and 28 days of cultivation using the NucleoSpin RNA Mini or XS kit (MACHEREY-NAGEL, Düren, Germany) following the manufacturer’s protocols. For every condition and sampling time, three wells were analyzed (n = 3 biological replicates for each donor). mRNA was quantified using the NanoDrop 2000c spectrophotometer (Thermo Fisher Scientific) and cDNA was synthesized using the TaqMan Reverse Transcription kit (Applied Biosystems, Foster City, USA) according to the manufacturer’s protocol. Real-time PCR was performed using the CFX96 real-time PCR system (Bio-Rad, Munich, Germany). For this, cDNA equivalent to 5 ng total mRNA, 10 µM of the respective primers and SensiFAST SYBR No-ROX qPCR master mix (Bioline, Luckenwalde, Germany) were mixed in a total volume of 20 µL. After each PCR run, a melting curve analysis was performed to exclude non-specific amplification. Relative mRNA expression of marker genes was normalized to the house-keeping gene ubiquitin-conjugating enzyme E2 D2 (*UBE2D2*).

**Table 1:** Sequences of primers used for real-time PCR.

Gene	Accession number	Description	For	Rev
*ALPL*	NM_000478	Alkaline phosphatase	cccacttcatctggaaccgc	ccgtggtcaattctgcctcc
*COL1A1*	NM_000088	Collagen type I alpha 1 chain	gccgtgacctcaagatgtg	gccgaaccagacatgcctc
*RUNX2*	NM_001015051	Runt-related transcription factor 2	tcacaaatcctccccaagtagc	ggcgggacacctactctcatac
*SPARC*	NM_003118	Secreted protein acidic and cysteine rich	gcagaagctgcgggtgaagaa	ctcgaaaaagcgggtggtgc
*SPP1*	NM_000582	Secreted phosphoprotein 1	cactgattttcccacggacct	ccattcaactcctcgctttcc
*UBE2D2*	NM_003339	Ubiquitin-conjugating enzyme E2 D2	tcttgacaattcatttcccaacag	tcaggcactaaaggatcatctgg

### Statistical analysis

Statistical analyses were performed using GraphPad Prism 8 (San Diego, USA). All values are given as mean ± s.d. One-way ANOVA with Tukey’s multiple comparison test was performed to analyze the overall impact of irradiation on the proliferation between donors. To investigate the impact of irradiation on proliferation for the combined data of all donors, two-way ANOVA with Tukey’s multiple comparison test was used. Brown-Forsythe ANOVA with Dunnett-T3 test was applied to analyze the proliferation data for significant changes between the irradiation conditions for each donor. For analysis of Real-time PCR data, two-way ANOVA with Tukey’s multiple comparison test was used. P values smaller than or equal to 0.05 were considered significant.

## Supplementary Information

Supplementary Information.

## Data Availability

The datasets generated during and/or analyzed during the current study are available from the corresponding author on reasonable request.
