# Supplementary material for: Pilot investigation on the dose-dependent impact of irradiation on primary human alveolar osteoblasts in vitro
Source: Sci Rep. 2021 Oct 6;11:19833. doi: 10.1038/s41598-021-99323-8 (PMC8494843; doi:10.1038/s41598-021-99323-8)
Supplement: Supplementary file 1 — Supplementary Information. [file 41598_2021_99323_MOESM1_ESM.pdf]

Pilot investigation on the dose-dependent impact of irradiation on primary human alveolar osteoblasts *in vitro*

Anna-Klara Amler[1,2]*, Domenic Schlauch[1,2], Selin Tüzüner[1,2], Alexander Thomas[1,2], Norbert Neckel[3], Ingeborg Tinhofer[4,5], Max Heiland[3], Roland Lauster[2], Lutz Kloke[1], Carmen Stromberger[4,†], Susanne Nahles[3,†]

[1]Cellbricks GmbH, Berlin, Germany

[2]Department of Medical Biotechnology, Technische Universität Berlin, Berlin, Germany

[3]Department of Oral and Maxillofacial Surgery, Charité-Universitätsmedizin Berlin, Corporate Member of Freie Universität Berlin, Humboldt Universität zu Berlin, Berlin Germany

[4]Department of Radiation Oncology, Charité-Universitätsmedizin Berlin, Corporate Member of Freie Universität Berlin, Humboldt-Universität zu Berlin, Berlin, Germany

[5]German Cancer Consortium (DKTK) partner site Berlin, Berlin, Germany

*Correspondence: Anna-Klara Amler – aka@cellbricks.com

† These authors contributed equally to this work.

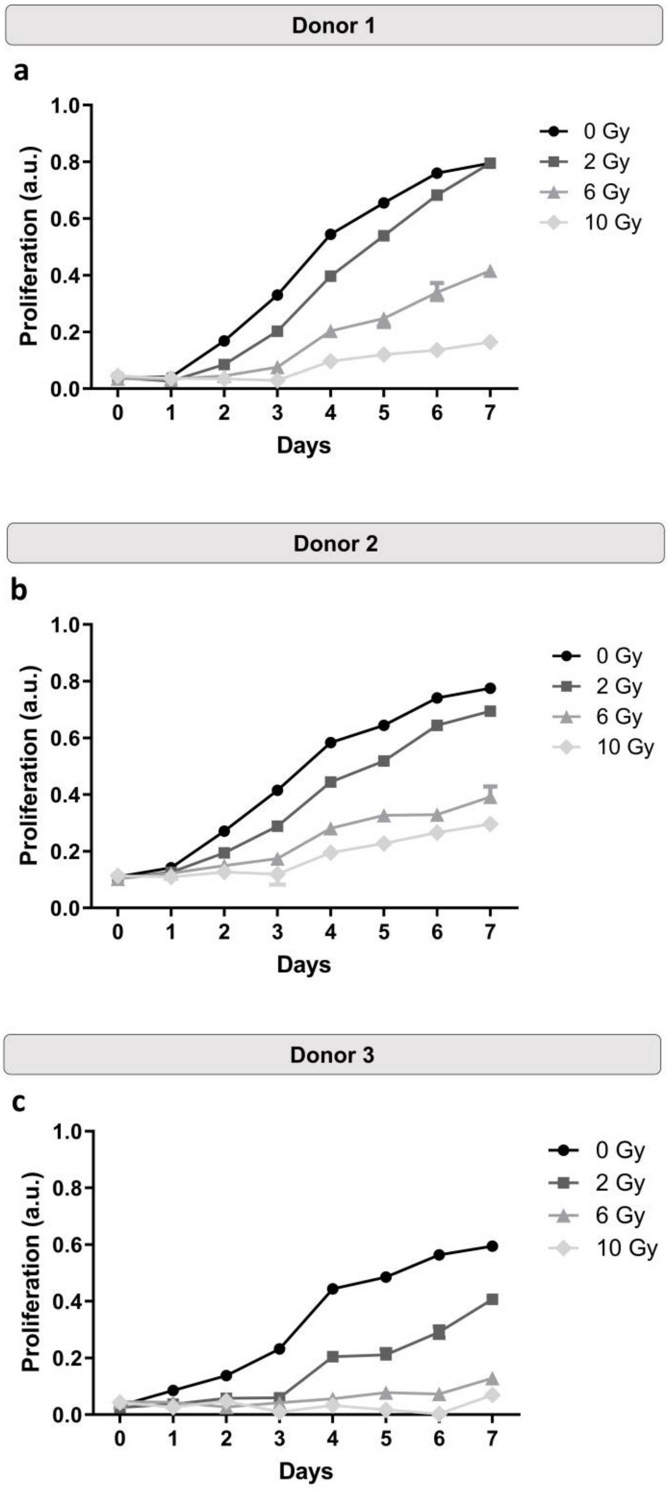

Supplementary Figure S1. Impact of irradiation on the proliferation of JHOBs. (a,b,c) Proliferation is inversely proportional to the MFI of the CellTrace Violet dye. JHOBs were stained with CellTrace Violet, seeded, and irradiated the next day. Sampling was performed every day and cells were analyzed using flow cytometry. Proliferation index is calculated as the inverse normalized MFI of CellTrace. Data are presented as mean ± s.d. n = 3 biological replicates for each donor.

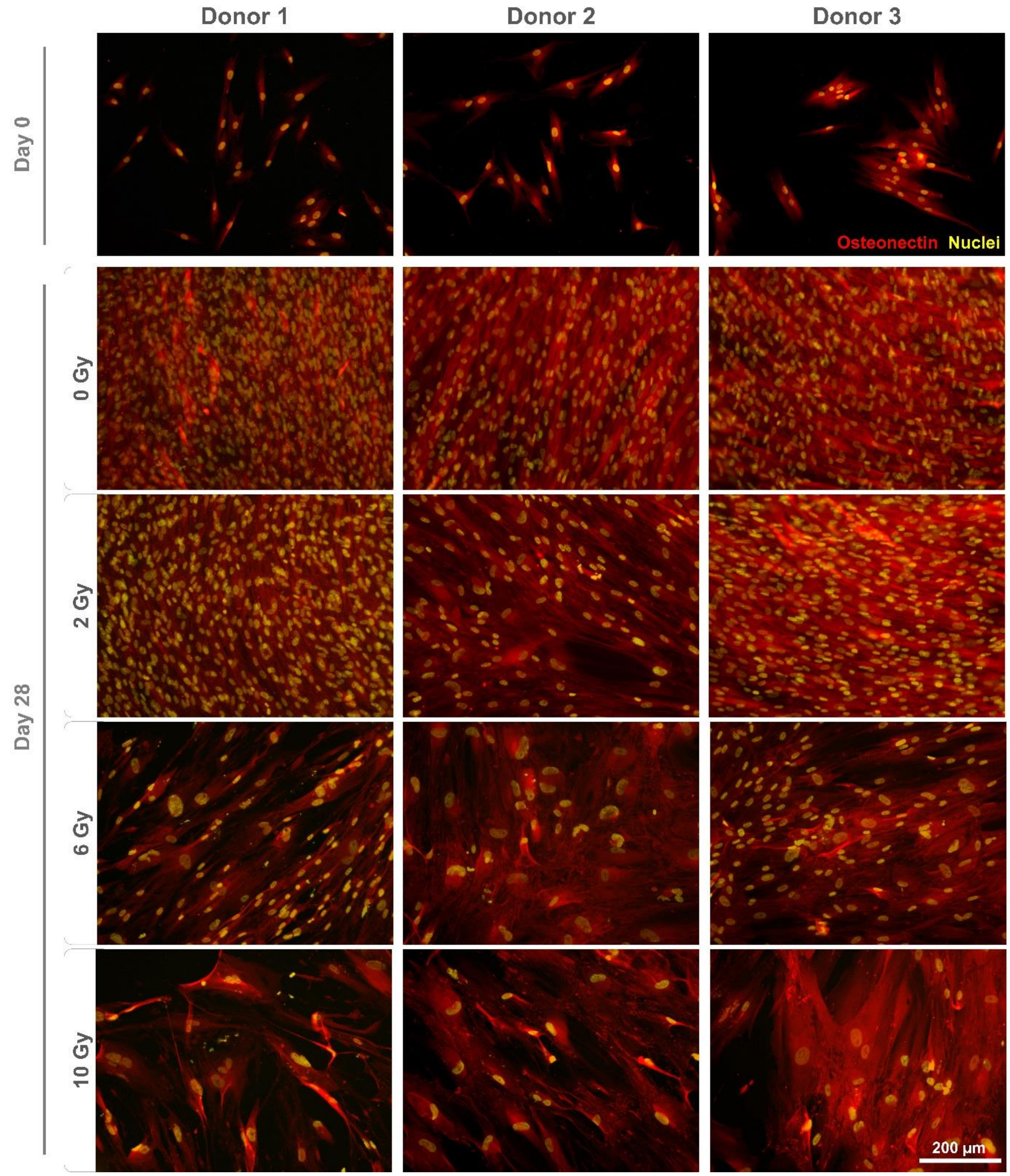

Supplementary Figure S2. Immunostaining of irradiated JHOBs for osteonectin. Cells were irradiated with 2, 6 and 10 Gy or sham, and stained for expression of osteonectin (red) after 28 days of cultivation in osteogenic medium. Non-treated cells on day 0 were used as a control. Nuclei were counterstained with DAPI (yellow).

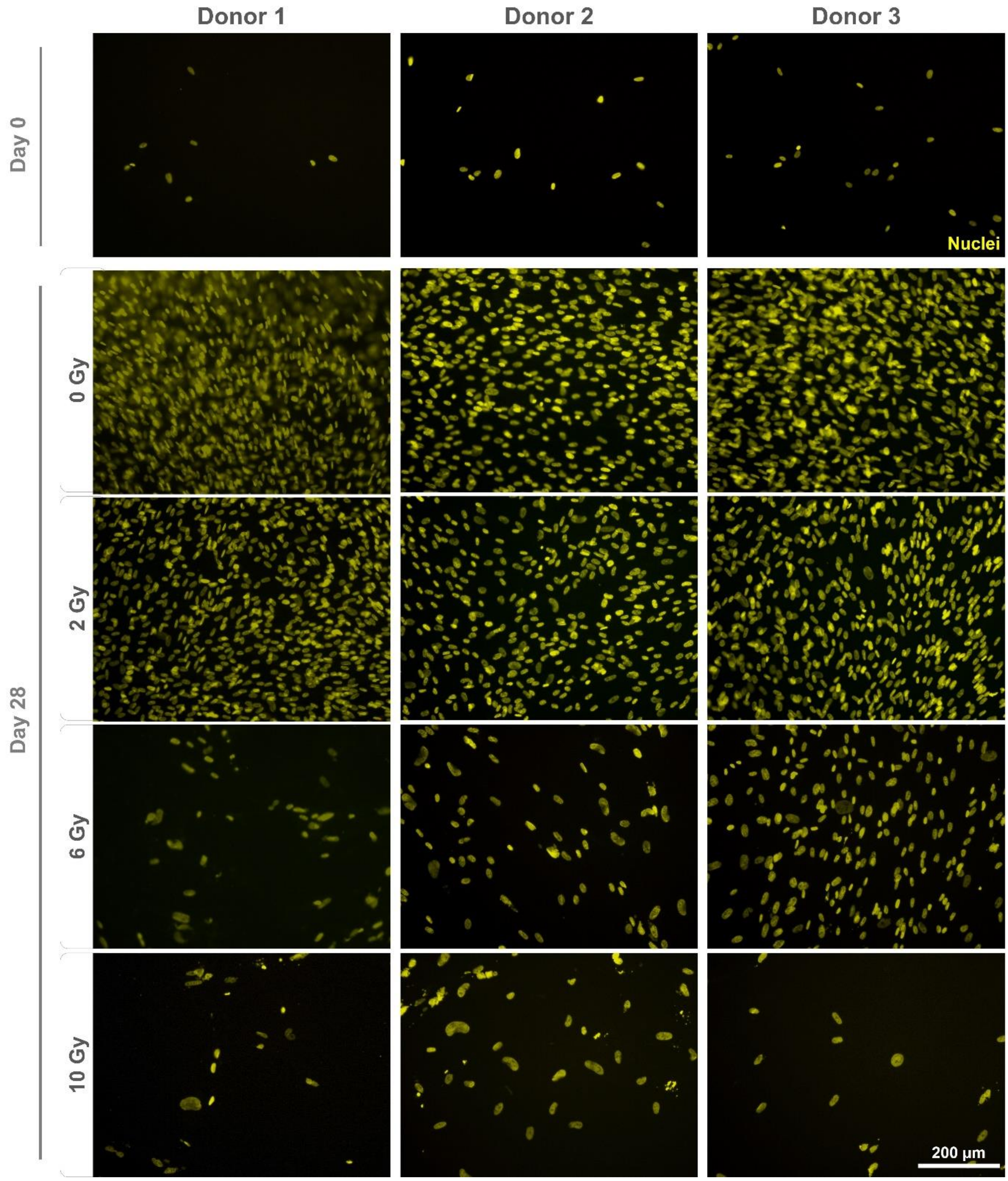

Supplementary Figure S3. Secondary antibody controls for the immunostaining of irradiated JHOBs. Cells were irradiated with 2, 6 and 10 Gy or sham, and stained with secondary antibodies after 28 days of cultivation in osteogenic medium to check for non-specific signals. Non-treated cells on day 0 were used as a control. Nuclei were counterstained with DAPI (yellow).

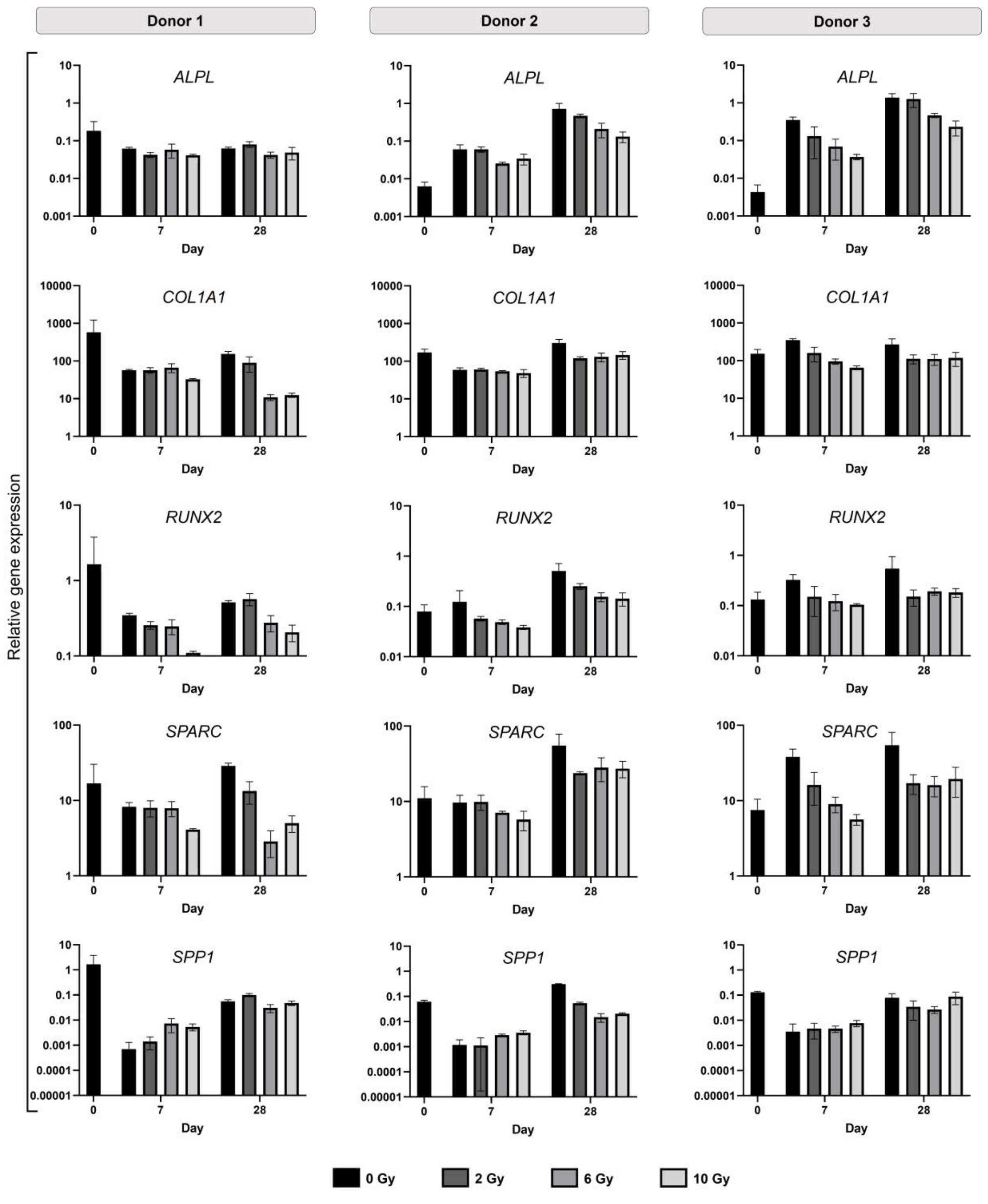

Supplementary Figure S4. **Marker gene expression of irradiated JHOBs for the individual donors.** Relative gene expression of the osteoblast differentiation markers *ALPL*, *COL1A1, RUNX2, SPARC*, and *SPP1* were cultivated in osteogenic medium and analyzed on days 0, 7 and 28. Expression was normalized to *UBE2D2* expression. Data are presented as mean ± s.d. n = 3 biological replicates for each donor.

Supplementary Table S1. Impact of irradiation on the proliferation of JHOBs (combined data of all donors). Two-way ANOVA of the proliferation index values with Tukey's multiple comparison test. Significant values (P < 0.05) are highlighted in bold.

	2 Gy	*6 Gy*	*10 Gy*
0 Gy	**<0.0001**	**<0.0001**	**<0.0001**
2 Gy		**<0.0001**	**<0.0001**
6 Gy			**0.0011**

Supplementary Table S2. Donor-dependent impact of irradiation on the proliferation of JHOBs. One-way ANOVA of the proliferation index values with Tukey's multiple comparison test. Significant values (P < 0.05) are highlighted in bold.

	P value
Donor 1 vs. donor 2	0.1454
Donor 1 vs. donor 3	**0.0011**
Donor 2 vs. donor 3	**<0.0001**

Supplementary Table S3. Impact of irradiation on the proliferation of JHOBs (separate data for each donor). Brown-Forsythe ANOVA of the proliferation index values with Dunnett-T3 test. Significant values (P < 0.05) are highlighted in bold.

Donor 1

	2 Gy	*6 Gy*	*10 Gy*
0 Gy	0.9519	**0.0065**	**<0.0001**
2 Gy		0.077	**0.0011**
6 Gy			**0.0379**

Donor 2

	2 Gy	*6 Gy*	*10 Gy*
0 Gy	0.7793	**0.002**	**0.0001**
2 Gy		**0.0437**	**0.0019**
6 Gy			0.2589

Donor 3

	2 Gy	*6 Gy*	*10 Gy*
0 Gy	**0.0219**	**<0.0001**	**<0.0001**
2 Gy		**0.0086**	**0.0005**
6 Gy			**0.002**

Supplementary Table S4. Marker gene expression of irradiated JHOBs (combined data of all donors). Two-way ANOVA with Tukey's multiple comparison test. Significant values (P < 0.05) are highlighted in bold.

ALPL	0 Gy	2 Gy	6 Gy	10 Gy
d0 vs. d7	0.6847	0.9753	>0.9999	0.9949
d0 vs. d28	**<0.0001**	**0.0002**	0.3192	0.7799
d7 vs. d28	**<0.0001**	**0.0002**	0.3013	0.7084

ALPL day 7	0 Gy	2 Gy	6 Gy	10 Gy
0 Gy	-	0.9171	0.8308	0.7746
2 Gy		**-**	0.9966	0.9887
6 Gy			**-**	0.9996
10 Gy				-

ALPL day 28	0 Gy	2 Gy	6 Gy	10 Gy
0 Gy	-	0.7924	**0.0014**	**<0.0001**
2 Gy		**-**	**0.0247**	**0.0021**
6 Gy			**-**	0.8531
10 Gy				-

COL1A1	0 Gy	2 Gy	6 Gy	10 Gy
d0 vs. d7	0.3776	0.1393	0.0943	0.0579
d0 vs. d28	0.8582	0.1821	0.1194	0.1381
d7 vs. d28	0.6971	0.9895	0.9932	0.9166

COL1A1 day 7	0 Gy	2 Gy	6 Gy	10 Gy
0 Gy	-	0.9382	0.8696	0.7607
2 Gy		**-**	0.9977	0.9779
6 Gy			**-**	0.9965
10 Gy				-

COL1A1 day 28	0 Gy	2 Gy	6 Gy	10 Gy
0 Gy	-	0.5958	0.4622	0.5060
2 Gy		**-**	0.9965	0.9990
6 Gy			**-**	0.9999
10 Gy				-

RUNX2	0 Gy	2 Gy	6 Gy	10 Gy
d0 vs. d7	0.5918	0.4058	0.3829	0.3041
d0 vs. d28	0.9605	0.6921	0.4927	0.4418
d7 vs. d28	0.7590	0.8864	0.9804	0.9641

RUNX2 day 7	0 Gy	2 Gy	6 Gy	10 Gy
0 Gy	-	0.9898	0.9853	0.9582
2 Gy		**-**	>0.9999	0.9974
6 Gy			**-**	0.9987
10 Gy				-

RUNX2 day 28	0 Gy	2 Gy	6 Gy	10 Gy
0 Gy	-	0.9467	0.8212	0.7758
2 Gy		**-**	0.9886	0.9774
6 Gy			**-**	0.9998
10 Gy				-

SPARC	0 Gy	2 Gy	6 Gy	10 Gy
d0 vs. d7	0.3534	0.9949	0.7209	0.3748
d0 vs. d28	**<0.0001**	0.4234	0.7174	0.5218
d7 vs. d28	**<0.0001**	0.3698	0.2723	**0.0438**

SPARC day 7	0 Gy	2 Gy	6 Gy	10 Gy
0 Gy	-	0.4533	0.1431	**0.0375**
2 Gy		**-**	0.9060	0.5987
6 Gy			**-**	0.9404
10 Gy				-

SPARC day 28	0 Gy	2 Gy	6 Gy	10 Gy
0 Gy	-	**<0.0001**	**<0.0001**	**<0.0001**
2 Gy		**-**	0.9637	0.9984
6 Gy			**-**	0.9893
10 Gy				-

SPP1	0 Gy	2 Gy	6 Gy	10 Gy
d0 vs. d7	0.2363	0.2181	0.2215	0.2223
d0 vs. d28	0.4658	0.3086	0.2484	0.2909
d7 vs. d28	0.8722	0.9751	0.9974	0.9852

SPP1 day 7	0 Gy	2 Gy	6 Gy	10 Gy
0 Gy	-	>0.9999	>0.9999	>0.9999
2 Gy		-	>0.9999	>0.9999
6 Gy			-	>0.9999
10 Gy				-

SPP1 day 28	0 Gy	2 Gy	6 Gy	10 Gy
0 Gy	-	0.9907	0.9723	0.9868
2 Gy		-	0.9991	>0.9999
6 Gy			-	0.9997
10 Gy				-
